# Explainable artificial intelligence in medical ultrasound: a WoSCC-based bibliometric analysis, evidence map, and Scopus concordance assessment

**DOI:** 10.3389/fmed.2026.1945642

**Published:** 2026-08-26

**Authors:** Zhenyu Shi, Zhen Hu, Chujun Wang, Chenyang Yu, Weiwei Zhang, Yuxin Luo, Chunquan Zhang

**Affiliations:** Department of Ultrasound, The Second Affiliated Hospital of Nanchang University, Nanchang, Jiangxi, China

**Keywords:** bibliometric analysis, clinical evaluation, evidence mapping, explainable artificial intelligence, external validation, medical ultrasound

## Abstract

**Background:**

Explainable artificial intelligence (XAI) is now used across medical ultrasound, yet it remains unclear whether the field has moved beyond producing explanation displays. This review examined whether the recent expansion of ultrasound XAI has been matched by explanation-specific evaluation and clinically relevant validation, and whether the leading bibliometric patterns are consistent across databases.

**Methods:**

Web of Science Core Collection (WoSCC) and Scopus were searched for records published from 2010 to 2026 and indexed through July 9, 2026. After cross-database deduplication, 1,745 unique records were assessed and database-specific Broad and nested Core XAI datasets were constructed. WoSCC supported bibliometric mapping and structured evidence coding; Scopus and union datasets were used to assess incremental coverage and cross-database concordance.

**Results:**

The searches retrieved 2,706 records and yielded 640 Union Broad and 514 Union Core XAI records; the primary WoSCC datasets contained 469 and 387 records, respectively. Publications rose sharply after 2023, with 2026 representing an incomplete year. SHAP (186/387, 48.1%) and CAM/Grad-CAM (79/387, 20.4%) predominated. An explanation output was reported or displayed without an identified evaluation procedure in 280/387 studies (72.4%), while no explicit explanation-evaluation information was identified in the reviewed evidence sources for 90/387 studies (23.3%); only 17 (4.4%) reported expert/reader, quantitative faithfulness or stability, or clinical-usefulness evaluation. External validation was reported in 62/387 records (16.0%), prospective design in 21/387 (5.4%), and multicenter design in 63/387 (16.3%). Country and cleaned-keyword ranks were highly concordant across databases (Spearman rho = 0.90 and 0.91), while Scopus added 36.5% Broad and 32.8% Core XAI records relative to WoSCC.

**Conclusion:**

Ultrasound XAI has expanded rapidly, but growth in the literature has not been matched by comparable maturity in reported explanation evaluation or clinically relevant validation. Progress will require testable explanation claims and evaluation under realistic acquisition and clinical conditions.

## 1 Introduction

Artificial intelligence (AI) is now used throughout medical imaging to combine image patterns with clinical variables and longitudinal information beyond what unaided visual assessment can readily integrate (1). In ultrasound, research has extended from lesion classification and detection to segmentation, measurement, risk prediction, report generation, and acquisition quality assessment. Breast ultrasound decision support has already been evaluated in multicohort and prospective settings (2), yet good predictive performance alone is not enough for clinical credibility. Models also need to perform across institutions, devices, operators, patient groups, and workflows, with outputs that inform rather than distort clinical reasoning (3–7).

Ultrasound creates a distinctive setting for explainability because images are produced through continuous interaction among the operator, probe, patient, and machine. Ultrasound images may vary across equipment, institutions, operators, patients, and acquisition settings, while artifacts can alter the information available to an algorithm (3–6). A heatmap that appears anatomically plausible on one stored frame may therefore change across adjacent frames or acquisition perturbations. Feature attribution in radiomics or clinical-tabular models can also be affected by correlated predictors, preprocessing, segmentation variability, and case mix. Displaying a SHAP plot, attention map, or class-activation map does not establish that the explanation faithfully represents model behavior or improves human decisions (8–15).

Medical-imaging XAI literature includes both reviews and experimental evaluation studies. Van der Velden et al. (13) catalogued available methods and proposed evaluation matched to clinical use, whereas Arun et al. (14) demonstrated weaknesses in saliency-map reliability. Subsequent systematic reviews highlighted limited clinician involvement in evaluation (15, 16), and an experimental framework quantified explanation quality in histopathology images (17). Bibliometric work has mapped AI activity in medical ultrasound (18). Ultrasound-focused reviews have examined oncological applications (19), while a broader healthcare XAI review considered imaging alongside diagnostic and rehabilitation applications (20). A breast-ultrasound review provided an organ-specific synthesis (21), and a second bibliometric analysis mapped AI research in the related carotid-imaging domain (22). What remains missing is a framework that connects the bibliometric structure of ultrasound XAI with record-level evidence on explanation evaluation and study validation. Such a framework must also separate database-specific science mapping from questions of retrieval coverage, because apparent leaders in countries, keywords, and growth may depend on the source searched.

This study used a systematic bibliometric design that combined cross-database analysis with a WoSCC-based evidence map of reported evaluation and validation. The main question was whether the rapid growth of ultrasound XAI has been accompanied by greater maturity in explanation evaluation and clinically relevant validation. Publication output, collaboration, conceptual structure, and intellectual foundations were described; the clinical domains, AI tasks, XAI methods, and evaluation status of the Core studies were mapped; and leading geographic and conceptual rankings were compared between WoSCC and Scopus together with the additional coverage provided by Scopus. Citation-derived analyses remained database specific, while union datasets were used only for coverage. This separation allowed database dependence to be examined without treating non-equivalent citation records as interchangeable.

## 2 Methods

### 2.1 Study design and reporting framework

This systematic bibliometric review combined performance analysis and science mapping with structured evidence coding and cross-database coverage assessment. The systematic bibliometric designation reflects systematic retrieval and quantitative bibliometric synthesis, whereas evidence mapping was an embedded analytic component. Reporting was informed by PRISMA 2020 (23), PRISMA-S (24), and applicable mapping items from PRISMA-ScR (25). The selection flow was adapted to the bibliographic-record unit of analysis, and no pooled clinical-effect estimate was undertaken.

Datasets were separated by analytic purpose. WoSCC Broad was used for bibliometric performance analysis and science mapping because complete cited-reference records and stable source-specific fields were available, and its nested Core XAI set supported structured evidence coding. Parallel Scopus datasets assessed incremental coverage and rank concordance, while union datasets described unique coverage after deduplication. Database-specific citation totals and citation-derived networks were not combined because WoSCC and Scopus differ in source coverage and field structure (26, 27).

### 2.2 Information sources and search strategies

WoSCC and Scopus were searched to cover clinical, imaging, and engineering journals. WoSCC supplied cited-reference fields for science mapping, whereas both databases supplied bibliographic and affiliation fields for retrieval, coverage assessment, and rank comparison. Eligible records were English-language articles and reviews published from 2010 to 2026 and indexed by July 9, 2026; conference proceedings were excluded.

The exact WoSCC Advanced Search query was: TS = ((“ultrasound” OR “ultrasonography” OR “echography” OR “ultrasonic imaging”) AND (“artificial intelligence” OR “deep learning” OR “machine learning” OR “neural network*”) AND (“explainable” OR “interpretable” OR “interpretability” OR “transparency” OR “black box” OR “saliency map” OR “heat map” OR “attention mechanism” OR “Grad-CAM” OR “SHAP” OR “LIME” OR “trustworthy AI”)). The exact Scopus search was: TITLE-ABS-KEY((“ultrasound” OR “ultrasonography” OR “echography” OR “ultrasonic imaging”) AND (“artificial intelligence” OR “deep learning” OR “machine learning” OR “neural network*”) AND (“explainable” OR “interpretable” OR “interpretability” OR “transparency” OR “black box” OR “saliency map” OR “heat map” OR “attention mechanism” OR “Grad-CAM” OR “SHAP” OR “LIME” OR “trustworthy AI”)) AND (LIMIT-TO(DOCTYPE,"ar") OR LIMIT-TO(DOCTYPE,"re")) AND LIMIT-TO(LANGUAGE,"English") AND PUBYEAR >2009 AND PUBYEAR <2027. The WoSCC search comprised an initial search through April 4, 2026, and an update covering April 5 through July 9, 2026, with the intervals defined by the WoSCC Index Date field; Full Record and Cited References were exported. Scopus was searched on July 9, 2026, and All Available Information including references was exported. The searches returned 1,076 WoSCC and 1,630 Scopus records. Export settings and yields are provided in Supplementary Table S1.

### 2.3 Record export and cross-database deduplication

Source exports were preserved unchanged, assigned stable identifiers, and standardized for comparison without modifying the raw files. Cross-database duplicate detection used normalized DOI, followed by exact or near-exact combinations of title, year, first author, journal, and other bibliographic identifiers. Candidate near-duplicates were reviewed before linkage. When records contained complementary metadata, the union representation retained both-source provenance while source-specific analyses used native records. A shared publication was counted once in the union set but could contribute once to each database-specific analysis. Deduplication and the WoSCC-only, shared, and Scopus-only components are summarized in Supplementary Table S2.

### 2.4 Eligibility criteria and analytic datasets

Records were eligible for the Broad definition when they reported original research or a review concerning AI or machine-learning methods applied to human medical ultrasound and included an interpretable, explainable, transparent, attention-based, attribution-based, feature-importance, concept-based, rule-based, or otherwise human-understandable component. Ultrasound had to be a substantive input, output, acquisition target, or clinical imaging context rather than an incidental mention. Reviews were eligible for the Broad landscape analyses but not for the Core XAI evidence sets. Across the WoSCC, Scopus, and Union datasets, Core XAI was restricted to original studies reporting an explicit XAI method, interpretable-by-design model, explanation output, or clearly articulated analysis of feature or region contribution. Conference abstracts, editorials, letters without substantive methods, non-human studies without direct medical-ultrasound relevance, non-ultrasound modalities without separable ultrasound content, and records lacking an AI or interpretability component were excluded.

The Core XAI definition was nested within Broad and required an explicit XAI method, interpretable-by-design model, explanation output, or clearly articulated analysis of feature or region contribution that could be assigned to the final coding taxonomy. Core was not treated as an independent additive corpus. Operational rules and dataset nesting are provided in Supplementary Table S2.

### 2.5 Screening and adjudication

All 1,745 unique bibliographic records were independently screened by two reviewers against the predefined eligibility criteria using titles, abstracts, keywords, document types, and other indexed fields. Disagreements were adjudicated by a third reviewer. Eligibility was assessed at the bibliographic-record level. Full texts were consulted only when indexed information was insufficient to determine eligibility or assign an evidence-coding category; this targeted consultation was not a separate universal full-text eligibility stage. The screening, adjudication, and analytic-set workflow is documented in Supplementary Table S2.

### 2.6 Bibliometric and network analyses

Bibliometric performance analysis described annual output, sources, document types, authorship, international coauthorship, country production, and citation indicators in the WoSCC Broad set. Country productivity and the single-country publication/multiple-country publication indicators followed the bibliometrix corresponding-author-country output derived from affiliation metadata. The country coauthorship network used all standardized author-affiliation countries with full counting. Rankings were ordered by descending count, with alphabetical ordering for exact count ties. These indicators were interpreted descriptively and were not used as direct measures of study quality. Science-mapping methods followed established bibliometric guidance (28). Analyses used R version 4.6.0, bibliometrix version 5.4.1 (29), igraph version 2.3.3, VOSviewer version 1.6.20 (30), CiteSpace version 6.4.R2 Advanced (64-bit) (31), and Python version 3.13.5. Full software versions, network parameters, coding rules, and thesauri are listed in Supplementary Table S3.

Author keywords were standardized using Keyword thesaurus, version 27 (v27), which merged spelling, number, hyphenation, abbreviation, and clearly synonymous variants while preserving clinically meaningful distinctions. The primary VOSviewer co-occurrence network used full counting, association-strength normalization, and a minimum occurrence threshold of five. A specific-topic sensitivity network excluded generic search terms and used a minimum occurrence threshold of four. Temporal prominence for complete years 2018–2025 displayed the nine most frequent non-generic author keywords, selected by total document occurrence in the complete-year WoSCC Broad set.

Reference co-citation analysis used the complete-year WoSCC subset for 2018–2025 (*n* = 302). CiteSpace version 6.4.R2 Advanced (64-bit) was configured with 1-year time slices, g-index selection (k = 25), Pathfinder, and Pruning Sliced Networks; clusters were labeled using the log-likelihood ratio algorithm. Citation bursts were generated in CiteSpace and re-rendered without recalculation. Cluster labels and bursts were interpreted as indicators of intellectual organization and citation attention rather than clinical importance.

### 2.7 Structured evidence coding

All 387 WoSCC Core XAI records represented original studies after verification of publication type. Each record received one mutually exclusive primary category for clinical domain, AI task, XAI method, explanation evaluation, and validation status. When several categories applied, the category most central to the main analysis or explanation claim was used. Categories and operational definitions are summarized in Table 1; Supplementary Table S3, with record-level coding provided in Supplementary Data S1.

**Table 1:** Evidence profile of the WoSCC core XAI set (*n* = 387).

Clinical domain	*n* (%)	AI task	*n* (%)	XAI method	*n* (%)
A. Clinical domains, AI tasks, and XAI methods					
Thyroid	82 (21.2)	Classification	102 (26.4)	SHAP	186 (48.1)
Breast	82 (21.2)	Detection	98 (25.3)	CAM/Grad-CAM	79 (20.4)
Other/unspecified	81 (20.9)	Measurement	61 (15.8)	Other or hybrid XAI methods	68 (17.6)
Other domains	47 (12.1)	Segmentation	59 (15.2)	Saliency/heatmap	19 (4.9)
Obstetric/fetal	26 (6.7)	Prognosis	56 (14.5)	Attention-based	15 (3.9)
Vascular	25 (6.5)	Report generation	6 (1.6)	Feature attribution	12 (3.1)
Liver	25 (6.5)	Quality assessment	4 (1.0)	Rules/knowledge	5 (1.3)
Cardiac	19 (4.9)	Other task	1 (0.3)	Prototype/concept	3 (0.8)
B. Explanation evaluation, validation status, and reported design attributes					
Explanation evaluation	*n* (%)	Validation status	*n* (%)	Study design attribute	*n* (%)
Explanation presented without identified evaluation	280 (72.4)	Not explicitly reported	208 (53.7)	Multicenter design reported	63 (16.3)
No explicit evaluation information identified	90 (23.3)	Internal validation	117 (30.2)	Prospective design reported	21 (5.4)
Expert/reader assessment	10 (2.6)	External validation	62 (16.0)		
Quantitative faithfulness/stability	4 (1.0)				
Clinical usefulness assessment	3 (0.8)				

Data are *n* (%). All 387 records represented original studies after verification of publication type. The column groups in each panel represent independent marginal distributions; entries aligned in the same row do not indicate an association. Each record contributes one primary category within each mutually exclusive evidence dimension. ‘Other domains’ comprises specified less-frequent domains, including lung, musculoskeletal, prostate, and gastrointestinal applications. ‘Other/unspecified’ comprises general, multidomain, or other records for which no single primary clinical domain could be assigned. Prospective and multicenter designs are reported separately as non-mutually exclusive attributes. Evaluation categories were assigned hierarchically. Records with an explicit evaluation procedure were assigned to the corresponding expert/reader, quantitative faithfulness or stability, or clinical-usefulness category. Among the remaining records, those with an identifiable explanation output were classified as ‘explanation presented without identified evaluation’; records for which neither a distinct explanation output nor an evaluation procedure could be identified in the reviewed sources were classified as ‘no explicit evaluation information identified.’ These labels do not establish definitive absence from unexamined full texts. XAI, explainable artificial intelligence.

Attention-based models were retained in the Core set only when attention outputs were explicitly presented or interpreted as explanations, or when attention formed part of a transparent decision mechanism whose contribution to the prediction could be traced. The presence of a performance-oriented attention module alone was insufficient for Core eligibility.

**Figure 1:**
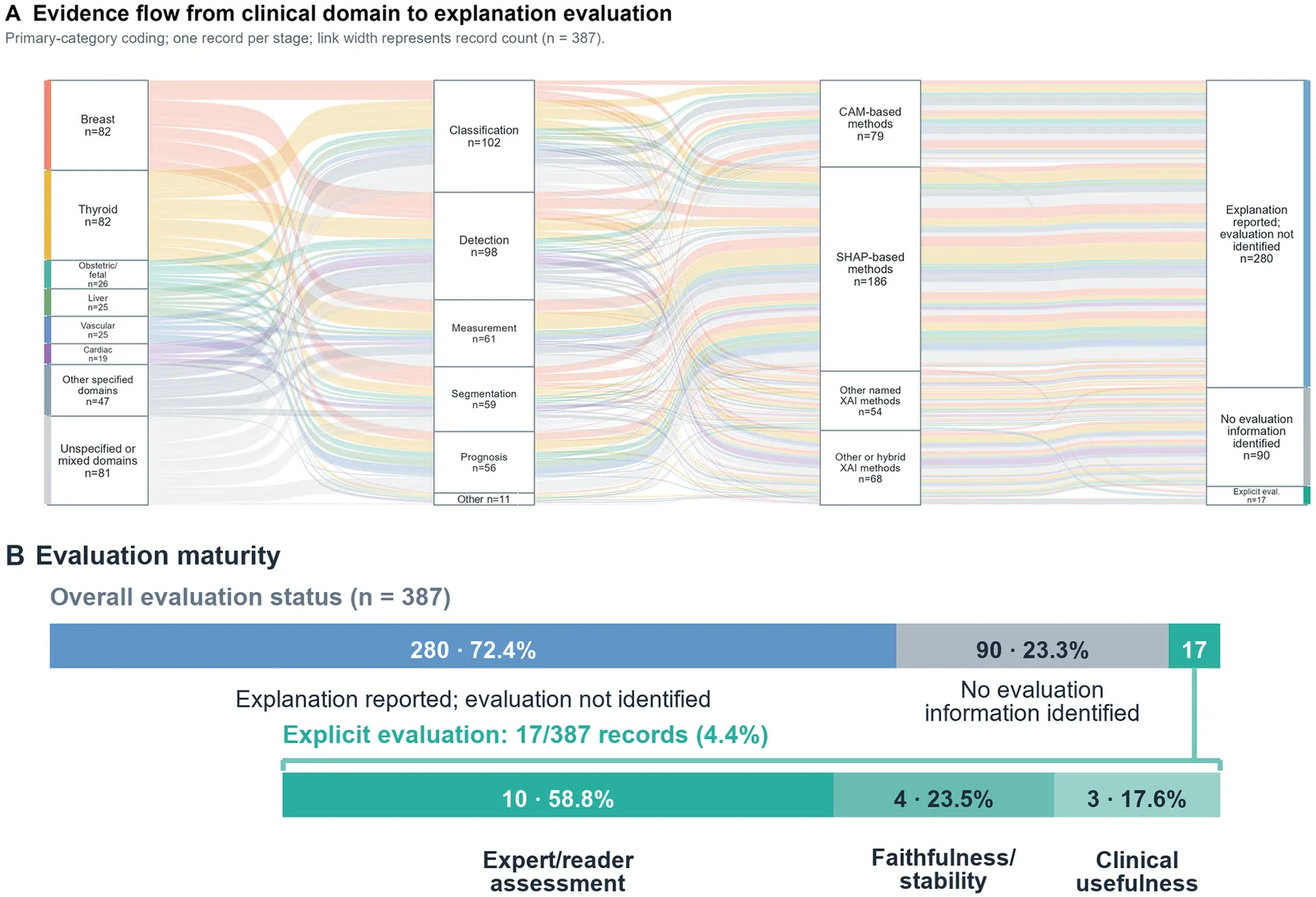
Evidence flow and reported explanation evaluation in the WoSCC core XAI dataset (*n* = 387). **(A)** Four-stage alluvial map linking each record's primary clinical domain, AI task, XAI method, and explanation-evaluation category; each record contributes one category at each stage, and link width represents record count. Other named XAI methods aggregated five identifiable low-frequency named families reported separately in Table 1 (*n* = 54), whereas other or hybrid XAI methods comprised multimethod, hybrid, or otherwise non-single-family approaches (*n* = 68). Evaluation categories were assigned hierarchically and were mutually exclusive. ‘Explanation reported; evaluation not identified’ indicates that an explanation output was identifiable but no distinct evaluation procedure was identified. ‘No evaluation information identified’ indicates that an XAI method or interpretable component qualified the record for the Core set, but neither a distinct explanation output nor an evaluation procedure could be identified in the reviewed sources. Neither label establishes definitive absence from an unexamined full text. **(B)** Overall reported explanation-evaluation categories and composition of the 17 records with explicit evaluation. Percentages use the 387-record WoSCC Core XAI dataset as the denominator unless otherwise specified. For display, “Other specified domains” corresponds to “Other domains” in Table 1, and “Unspecified or mixed domains” corresponds to “Other/unspecified”.

Other or hybrid XAI methods comprised multimethod, hybrid, or otherwise non-single-family approaches. For display in Figure 1, the five low-frequency named families reported separately in Table 1—saliency or heatmap, attention-based, other feature-attribution, rule- or knowledge-based, and prototype- or concept-based methods—were aggregated as ‘Other named XAI methods’ (*n* = 54). This display category was distinct from ‘Other or hybrid XAI methods’ (*n* = 68).

Evaluation categories were assigned hierarchically and were mutually exclusive. Records with an explicit expert or reader assessment, quantitative faithfulness or stability test, or clinical-usefulness evaluation were assigned to the corresponding evaluation category. Among the remaining records, those with an identifiable explanation output were classified as ‘explanation presented without identified evaluation.’ Records in which an XAI method or interpretable component qualified the study for the Core set but neither a distinct explanation output nor an evaluation procedure could be identified in the reviewed sources were classified as ‘no explicit evaluation information identified.’ Expert or reader evaluation required explicit human assessment; quantitative evaluation required a numerical test of model linkage, localization, repeatability, reproducibility, sensitivity, or robustness; and clinical usefulness required a workflow-relevant outcome. These categories describe information identified in the reviewed evidence sources and do not establish definitive absence from unexamined full texts.

Validation status was coded as not explicitly reported, internal validation, or external validation on the basis of information identified in the reviewed sources. External validation required clearly reported evaluation in an independently sourced cohort, institution, geographic setting, or other external dataset beyond a routine split from the development source. Prospective and multicenter designs were coded separately as non-mutually exclusive reported attributes.

### 2.8 Cross-database concordance and incremental coverage analysis

Cross-database concordance was assessed using harmonized database-specific datasets. Normalized annual publication profiles for complete years 2018–2025 were compared descriptively; no correlation coefficient was used for these short, strongly increasing time series. Corresponding-author-country rank concordance included countries appearing among the top 20 in both database-specific Broad sets after country-name standardization (*n* = 18). Corresponding-author country was available for 469/469 WoSCC and 591/611 Scopus records. Cleaned author-keyword concordance included terms appearing among the top 25 in both Broad sets after application of the same v27 thesaurus (*n* = 22); author keywords were available for 441/469 and 576/611 records, respectively. Missing fields were not imputed. Shared-list ranks were compared using Spearman correlations with 2,000-resample bootstrap 95% confidence intervals. Top-list overlap and Jaccard indices were also calculated. For each entity appearing in the union of the database-specific leading lists, its rank in the complete database-specific ranking was used, and Spearman correlations were recalculated across the union of leading entities. Paired WoSCC and Scopus ranks were displayed for each shared country or keyword, with connecting lines indicating database-specific rank differences. Concordance was interpreted as a descriptive sensitivity analysis of database choice, not as independent external validation.

Incremental Scopus coverage was calculated from mutually exclusive union components as Scopus-only records divided by the corresponding WoSCC set. Rank-comparison variables are reported in Supplementary Table S5, with public record-level provenance for the Scopus-only component in Supplementary Data S2.

### 2.9 Statistical analysis and reproducibility

Continuous and count variables were summarized descriptively. Percentages used the analysis-specific denominator and were reported to one decimal place where appropriate. Spearman rank correlation was used for country and keyword rankings because ranks were the estimand and normality was not assumed. Bootstrap 95% confidence intervals for rank correlations used 2,000 resamples, with countries and keywords as the respective resampling units. Cross-database concordance was interpreted descriptively rather than as a formal hypothesis test.

Because the Core studies encompassed heterogeneous tasks and designs, no single design-specific risk-of-bias tool was applicable. Evidence maturity was instead described using four predefined indicators: validation status, explanation evaluation, prospective design, and multicenter design. These indicators were not treated as a formal risk-of-bias assessment.

Analysis inputs, codebooks, software parameters, Keyword thesaurus, version 27 (v27), and derived source data were retained. A curated reproducibility package containing public Python reconstruction scripts, software parameters and logs, version-27 thesauri, and non-proprietary derived source data is provided as Supplementary Data S3; licensed WoSCC and Scopus exports are not included. Generative AI was used solely for English-language editing. All scientific content, analyses, interpretations, and final wording were reviewed and approved by the authors.

## 3 Results

### 3.1 Record selection and analytic datasets

**Figure 2:**
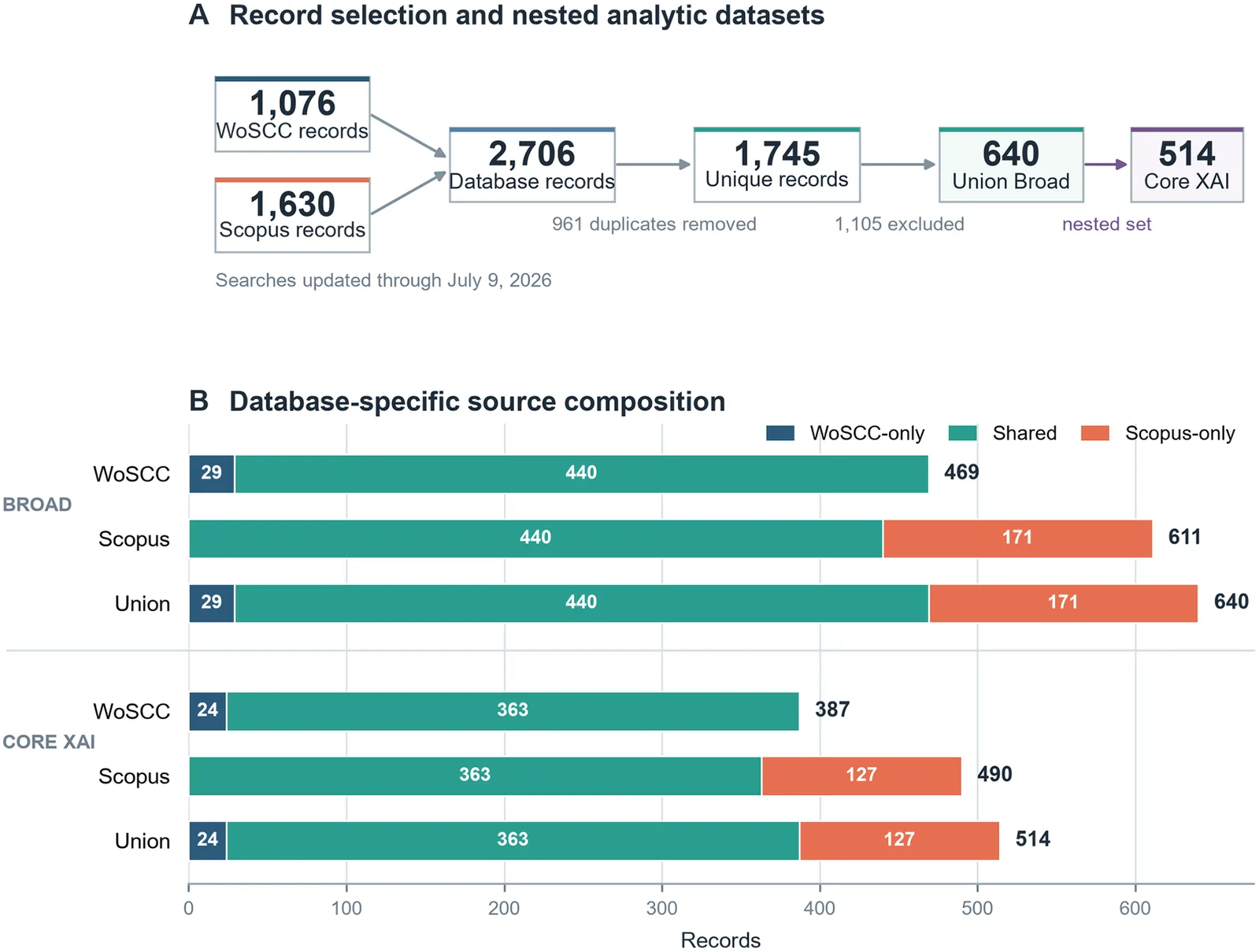
Record selection, database roles, and construction of the analytic datasets. **(A)** Records retrieved from Web of Science Core Collection (WoSCC) and Scopus, cross-database deduplication, eligibility assessment, and construction of the Union Broad set and its nested Union Core XAI set. Searches were updated through July 9, 2026. Eligibility was assessed primarily from indexed bibliographic fields, with targeted full-text consultation when required. **(B)** Mutually exclusive database-source components of the WoSCC, Scopus, and Union Broad and Core XAI sets. Core XAI is nested within Broad.

The searches identified 2,706 records: 1,076 from WoSCC and 1,630 from Scopus. Cross-database linkage removed 961 duplicates, leaving 1,745 unique records for eligibility assessment. After 1,105 exclusions, 640 records met the Broad definition and 514 met the nested Core XAI definition (Figure 2A). Core XAI represented 80.3% of Union Broad, while Broad-only records contributed to the wider publication and intellectual landscape.

Union Broad comprised 440 shared, 29 WoSCC-only, and 171 Scopus-only records; Union Core XAI comprised 363 shared, 24 WoSCC-only, and 127 Scopus-only records (Figure 2B). The database-specific sets were WoSCC Broad *n* = 469 and Core XAI *n* = 387, and Scopus Broad *n* = 611 and Core XAI *n* = 490.

### 3.2 Publication landscape and collaboration

**Figure 3:**
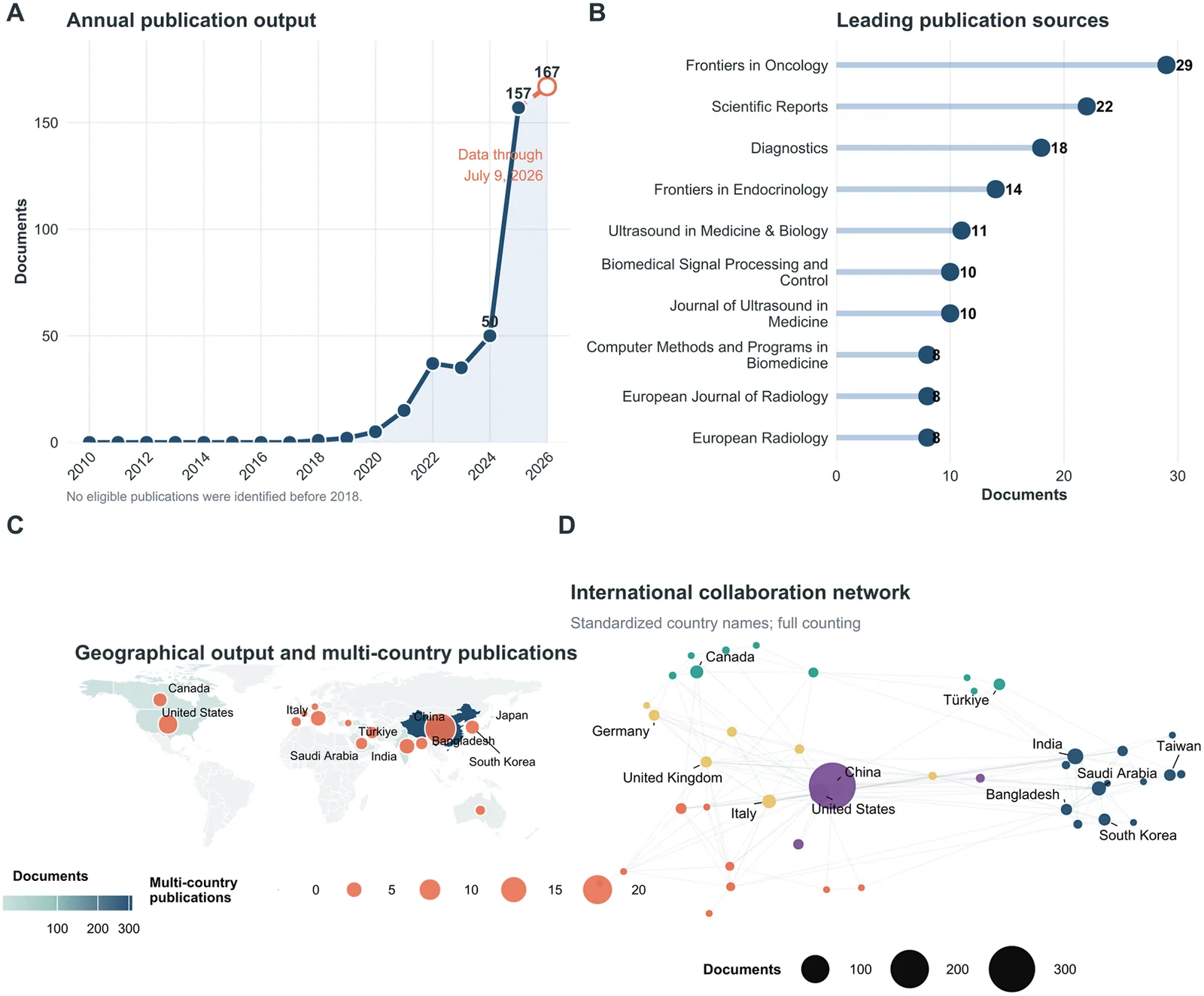
Publication landscape and international collaboration in the WoSCC broad set. **(A)** Annual publication output for publication years 2010–2026; 2026 data reflect records indexed through July 9, 2026. The hollow point and dashed final segment identify partial-year 2026, which was excluded from complete-year temporal inference. **(B)** Ten leading publication sources. **(C)** Corresponding-author-country output; shading represents document volume and circle size represents multi-country publications. **(D)** International co-authorship network using all standardized author-affiliation countries with full counting. Node size represents document volume, edge weight collaboration strength, and node color the VOSviewer cluster.

The WoSCC Broad set contained 469 documents from 191 sources across publication years 2018–2026; 2026 records were indexed through July 9, 2026, and no eligible records were identified from 2010 through 2017. Annual output was initially sparse, with one record in 2018, two in 2019, five in 2020, and 15 in 2021. Output increased to 37 in 2022, 35 in 2023, 50 in 2024, and 157 in 2025. The 167 records with publication year 2026 represent an incomplete-year snapshot indexed through July 9, 2026 and were excluded from complete-year trend inference (Figure 3A).

Articles accounted for 451 records (96.2%) and reviews for 18 (3.8%). The corpus contained 16,240 cited references, 1,204 author keywords, and 592 Keywords Plus terms. It involved 2,817 authors, with a mean of 7.47 coauthors per document; 17.7% of documents involved international coauthorship. Mean citations were 9.47 per document, although this measure is sensitive to publication age and database coverage (Table 2).

**Table 2:** Main bibliometric characteristics of the WoSCC broad set (*n* = 469).

Characteristic	Result	Characteristic	Result
Timespan	2018–2026	Sources	191
Documents	469	Cited references	16,240
Articles, *n* (%)	451 (96.2)	Reviews, *n* (%)	18 (3.8)
Author keywords	1,204	Keywords Plus	592
Authors	2,817	Author appearances	3,505
Single-authored documents	4	Co-authors per document	7.47
International co-authorship rate, %	17.7	Average citations per document	9.47

Values are based on the WoSCC Broad set. Data for 2026 were available through July 9, 2026. Article and review counts include early-access records. WoSCC, Web of Science Core Collection.

The leading sources were Frontiers in Oncology (29 documents), Scientific Reports (22), Diagnostics (18), Frontiers in Endocrinology (14), and Ultrasound in Medicine & Biology (11), within a broader distribution spanning clinical, imaging, and engineering journals (Figure 3B).

China contributed 311 corresponding-author-country documents, followed by the United States (18), India (16), and Canada (14). China produced 290 single-country and 21 multi-country publications, while the United States produced 10 and eight, respectively. The collaboration network, which used all author-affiliation countries with full counting, was centered on the dominant high-output country with smaller international links (Figures 3C,D). These two country indicators are not interchangeable and do not measure clinical implementation or population representativeness. Detailed source, author, country, and keyword rankings are provided in Supplementary Table S4.

### 3.3 Conceptual structure and temporal evolution

**Figure 4:**
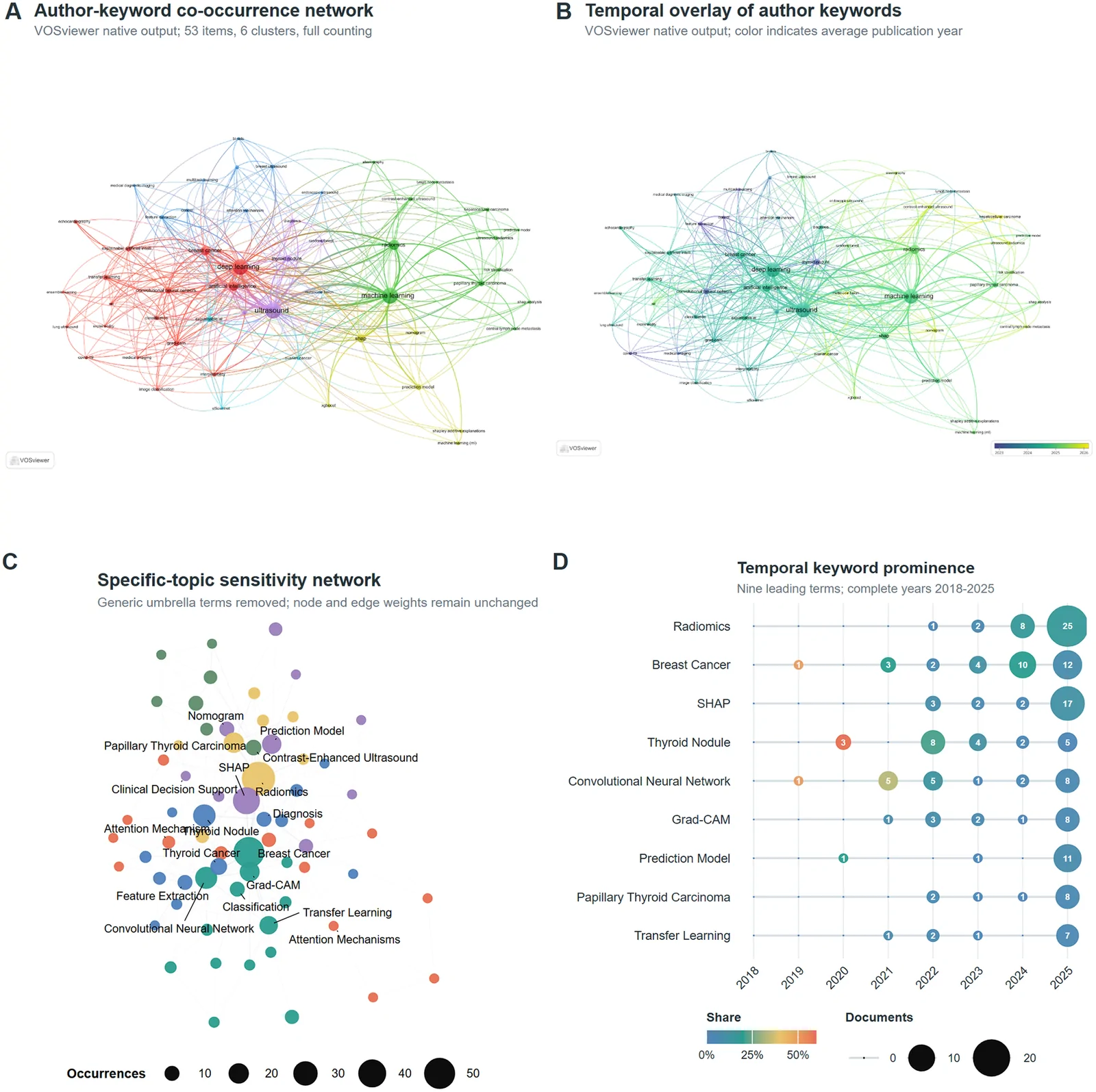
Conceptual structure and temporal prominence of ultrasound XAI research. **(A)** Native VOSviewer author-keyword co-occurrence network after application of Keyword thesaurus, version 27 (v27), using full counting and a minimum occurrence threshold of five. **(B)** Native VOSviewer temporal overlay of the same network; node color represents average publication year. **(C)** Specific-topic sensitivity network after removal of generic search terms, using a minimum occurrence threshold of four. **(D)** Temporal prominence of the nine most frequent non-generic author keywords from 2018 to 2025. Terms were selected by total document occurrence in the complete-year WoSCC Broad set after removal of generic search terms. Bubble size and labels represent document count, and color represents within-year share.

After application of Keyword thesaurus, version 27 (v27), 53 author keywords met the minimum occurrence threshold of five. The final VOSviewer network contained six clusters, 361 links, and total link strength of 1,127 (Figure 4A). “Machine learning” (154 occurrences), “ultrasound” (152), and “deep learning” (140) formed the central vocabulary. These general terms linked clinical application nodes to method-specific terms, indicating that the field is organized around a broad AI-ultrasound core rather than around isolated disease-specific literatures.

Recent terms included “radiomics” (58 occurrences; average publication year 2025.12), “artificial intelligence” (53; average year 2024.60), “breast cancer” (49), “explainable artificial intelligence” (44), and “SHAP” (37; average year 2024.95). Thyroid nodule (25), convolutional neural network (24), papillary thyroid carcinoma (20), Grad-CAM (19), prediction model (18), and transfer learning (16) were also recurrent. The temporal overlay placed radiomics and SHAP toward the recent end of the network, whereas deep learning and ultrasound occupied its longer-standing center (Figure 4B).

The specific-topic sensitivity network, which excluded generic search terms, retained the principal clinical and method-specific structure (Figure 4C). The complete-year prominence display of the nine most frequent non-generic author keywords showed accelerating counts in 2024 and 2025 (Figure 4D). Bubble size represents document count and color represents within-year share. This expansion of XAI vocabulary does not establish more rigorous clinical evaluation.

### 3.4 Intellectual foundations and citation dynamics

**Figure 5:**
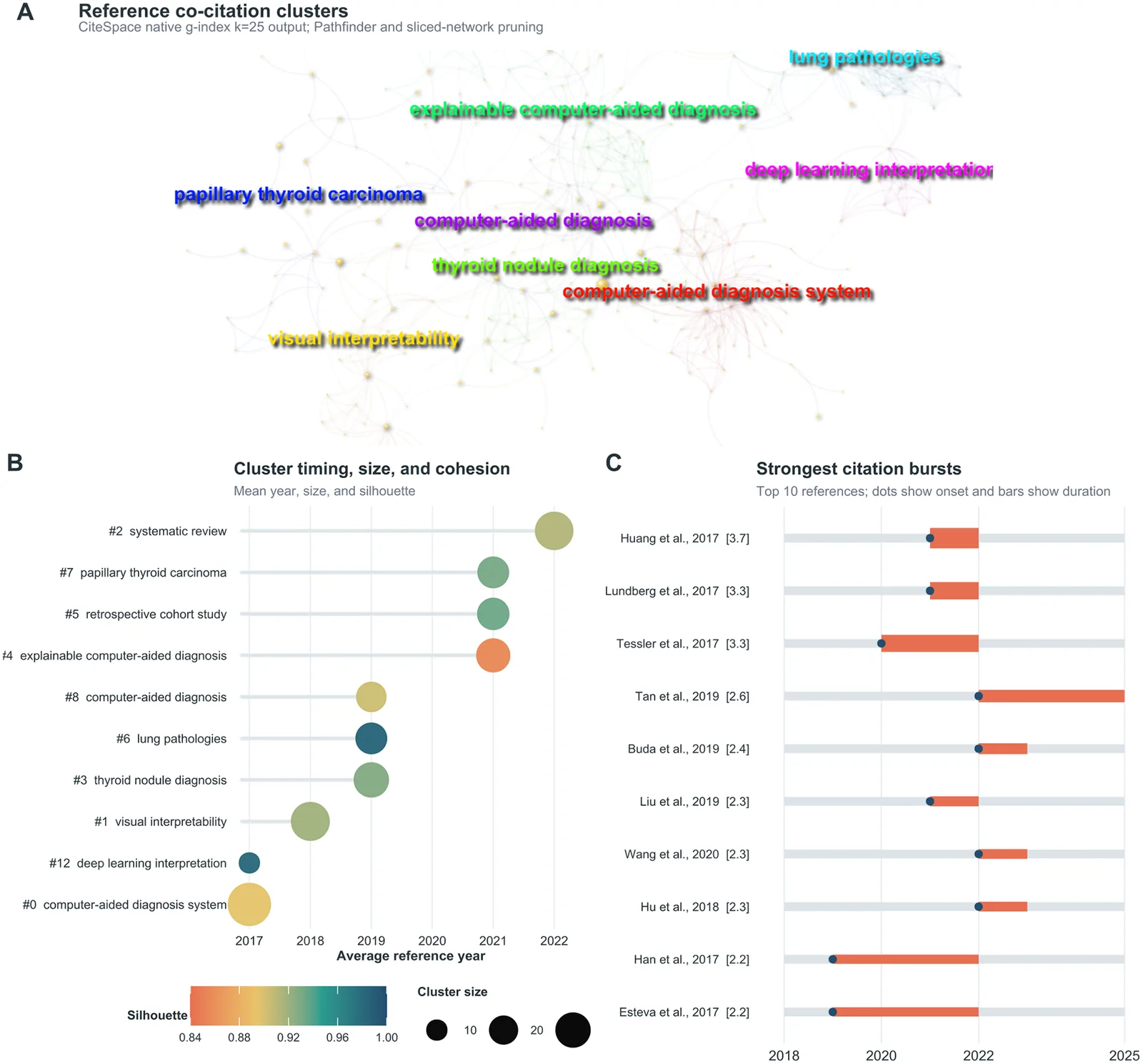
Intellectual foundations and citation dynamics. **(A)** Native CiteSpace reference co-citation cluster map using g-index selection (k = 25), Pathfinder, and Pruning Sliced Networks. **(B)** Cluster mean reference year, size, and cohesion using CiteSpace-exported cluster size, mean reference year, silhouette, and log-likelihood ratio labels; cluster identifiers and labels are identical to panel A. **(C)** Ten references with the strongest citation bursts. Gray lines show the observation window, colored segments show burst intervals, blue points show burst onset, and bracketed values show CiteSpace burst strength. Burst parameters were re-rendered in R without recalculation.

The 2018–2025 reference co-citation network formed 10 clusters (modularity Q = 0.807; weighted mean silhouette = 0.912), with 282 of 365 nodes in the largest connected component. The largest clusters were labeled computer-aided diagnosis system (#0; size 46), visual interpretability (#1; size 37), systematic review (#2; size 36), thyroid nodule diagnosis (#3; size 30), and explainable computer-aided diagnosis (#4; size 28). Additional clusters represented retrospective cohorts, lung pathologies, papillary thyroid carcinoma, and deep-learning interpretation (Figures 5A,B).

The structure connected an early foundation of deep-learning architectures and computer-aided diagnosis, a disease-specific application layer, and a newer layer of visual interpretability, SHAP-based attribution, and explainable computer-aided diagnosis. XAI methods therefore appeared within an established diagnostic AI literature rather than as a separate evidence tradition.

The strongest reference bursts further reflected this sequence (Figure 5C). DenseNet had the highest burst strength (3.721; 2021–2022) (32), followed by the SHAP framework (3.303; 2021–2022) (33), ACR TI-RADS (3.273; 2020–2022) (34), EfficientNet (2.638; 2022–2025) (35), and a thyroid-ultrasound deep-learning study (2.434; 2022–2023) (36). Other bursts involved thyroid diagnostic AI, squeeze-and-excitation networks (37), deep-learning interpretation, and broader medical-AI literature (38). EfficientNet was the only one of the ten strongest bursts extending through 2025. Burst strength represents concentrated citation attention within the analyzed network, not comparative clinical validity. Detailed cluster and burst results are provided in Supplementary Table S4.

### 3.5 XAI methods and evaluation maturity

The WoSCC Core XAI evidence map contained 387 original studies after verification of publication type. Thyroid and breast applications were the largest primary clinical domains, each with 82 records (21.2%). Other or unspecified applications accounted for 81 (20.9%), other named domains for 47 (12.1%), obstetric or fetal applications for 26 (6.7%), vascular and liver applications for 25 each (6.5%), and cardiac applications for 19 (4.9%). ‘Other named domains’ comprised specified domains too infrequent for separate display; ‘Other/unspecified’ comprised general, multidomain, or other records for which no single primary clinical domain could be assigned.

Classification was the leading primary task (102/387, 26.4%), followed by detection (98/387, 25.3%), measurement (61/387, 15.8%), segmentation (59/387, 15.2%), and prognosis or risk prediction (56/387, 14.5%). Report generation (6/387, 1.6%), quality assessment (4/387, 1.0%), and other tasks (1/387, 0.3%) were uncommon.

**Figure 6:**
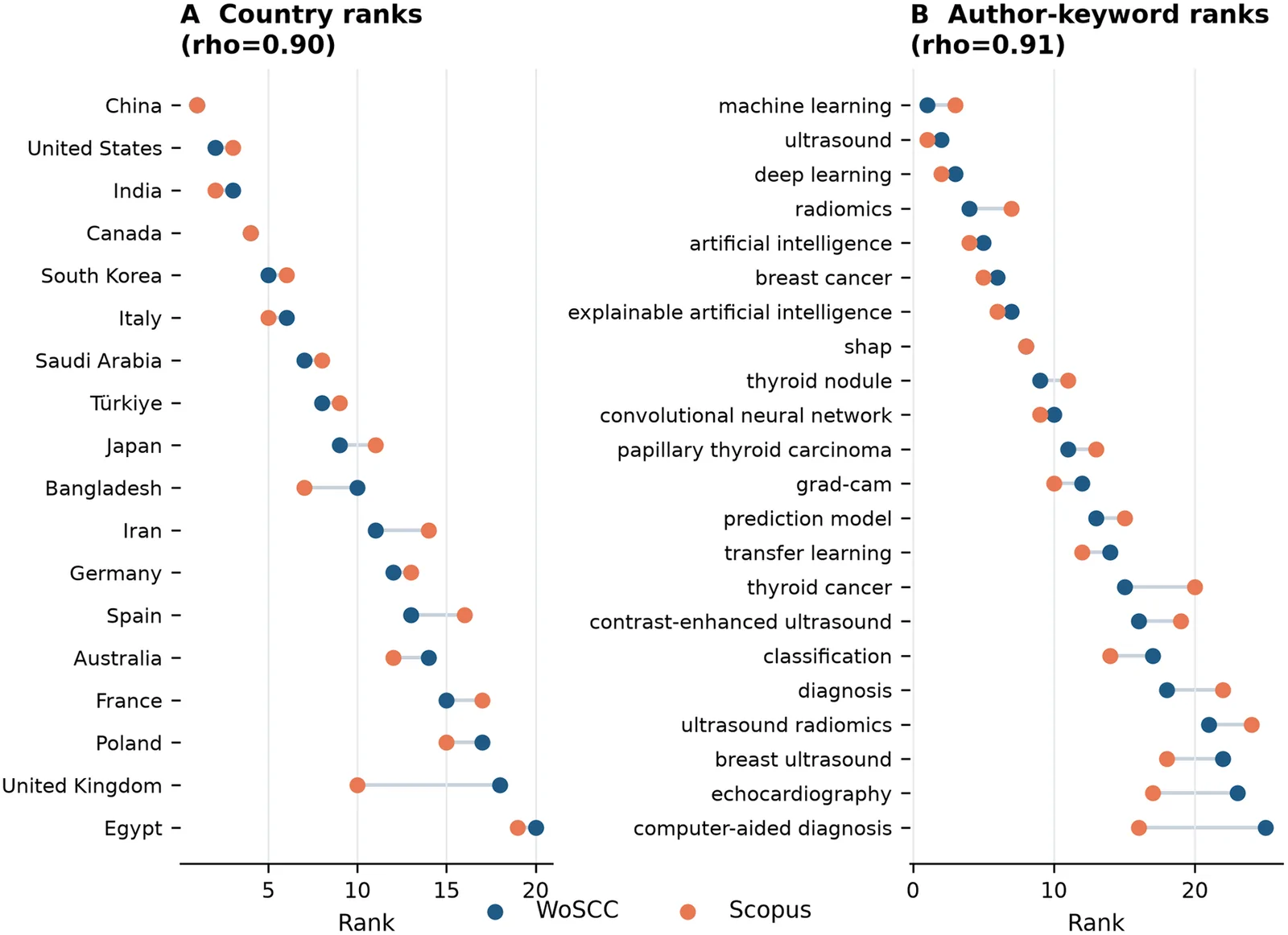
Cross-database rank concordance. **(A)** Concordance of corresponding-author-country publication ranks among countries appearing in the top 20 of both datasets (*n* = 18). **(B)** Concordance of cleaned author-keyword ranks among terms appearing in the top 25 of both datasets after applying the same thesaurus (*n* = 22). Spearman correlations with 2,000-resample bootstrap 95% confidence intervals are reported for panels A and B. Supplementary Table S5 reports top-list overlap and union-list sensitivity analyses.

SHAP was the most frequently coded XAI method (186/387, 48.1%), followed by CAM/Grad-CAM (79/387, 20.4%). Other or hybrid XAI methods accounted for 68 (17.6%), saliency or heatmap approaches for 19 (4.9%), attention mechanisms for 15 (3.9%), other feature-attribution methods for 12 (3.1%), rule- or knowledge-based approaches for five (1.3%), and prototype- or concept-based approaches for three (0.8%). Figure 6A traces the primary clinical domain, AI task, XAI method, and evaluation category assigned to each Core record; Supplementary Figure S1A shows the clinical-domain-by-method distribution.

An explanation output was reported or displayed without an identified evaluation procedure in 280 records (72.4%), whereas no explicit explanation-evaluation information was identified in the reviewed evidence sources for 90 (23.3%). Expert or reader evaluation was reported in 10 records (2.6%), quantitative faithfulness or stability testing in four (1.0%), and clinical-usefulness evaluation in three (0.8%) (Figure 6B).

External validation was identified in the reviewed sources for 62 records (16.0%), internal validation for 117 (30.2%), and no explicit validation status for 208 (53.7%). Multicenter design was reported in 63 records (16.3%) and prospective design in 21 (5.4%); these attributes were coded independently and could overlap (Supplementary Figure S1B). Reported explanation-specific evaluation was uncommon in every major clinical domain. Table 1 provides the complete category counts and percentages, and Supplementary Data S1 contains the concise record-level coding.

### 3.6 Cross-database concordance and incremental coverage

Normalized annual publication profiles for 2018–2025 showed the same transition from sparse early activity to rapid recent growth in WoSCC and Scopus (Supplementary Figure S2). The short, strongly time-dependent series were compared descriptively.

Country publication ranks were highly concordant across databases (Spearman rho = 0.9009; *n* = 18; bootstrap 95% CI, 0.6592–0.9832) (Figure 6A). Cleaned author-keyword ranks were similarly concordant (rho = 0.9119; *n* = 22; bootstrap 95% CI, 0.7255–0.9648) (Figure 6B). The top-list overlaps were 18/20 countries (90.0%; Jaccard, 81.8%) and 22/25 keywords (88.0%; Jaccard, 78.6%). In union-list sensitivity analyses using complete database-specific ranks, concordance remained high for countries (rho = 0.9085; *n* = 22) and keywords (rho = 0.8851; *n* = 28). These estimates describe agreement in the leading rankings, not equivalence of absolute counts or citation networks.

Scopus nevertheless provided substantial incremental coverage. Relative to the 469-record WoSCC Broad set, 171 Scopus-only records increased the union by 36.5%. Relative to the 387-record WoSCC Core XAI set, 127 Scopus-only records increased the union by 32.8% (Supplementary Figure S2B). The Core increment shows that Scopus added records meeting the explicit XAI definition as well as broader AI-ultrasound literature. Table 3 summarizes the source decomposition and incremental yields. Supplementary Table S5 and Supplementary Data S2 provide the comparison variables and record-level provenance.

**Table 3:** Cross-database coverage and incremental yield of Scopus.

Analytic set	WoSCC	Scopus	Shared	WoSCC-only	Scopus-only	Union	Relative Scopus-only yield, %
Broad	469	611	440	29	171	640	36.5
Core XAI	387	490	363	24	127	514	32.8

Broad and Core XAI denote predefined nested analytic sets. Relative Scopus-only yield was calculated against the corresponding WoSCC set (171/469 for Broad and 127/387 for Core XAI). Shared records were identified after cross-database deduplication. WoSCC, Web of Science Core Collection.

## 4 Discussion

### 4.1 Principal findings

Ultrasound XAI expanded rapidly, but reported evaluation lagged behind method adoption. Only 17 of 387 Core studies reported evaluation beyond explanation presentation, and external validation, multicenter design, and prospective evaluation remained uncommon. WoSCC and Scopus produced similar leading rankings, although Scopus added substantial unique coverage.

### 4.2 Publication growth vs. evaluation maturity

Publication output accelerated after 2023. SHAP and Grad-CAM provide established *post hoc* approaches for feature attribution and convolutional-network localization, respectively (33, 39). Their widespread use has made explanation displays a prominent feature of the literature, but the intended explanation claim and its evaluation were often less clearly developed.

Predictive validation and explanation evaluation answer different questions. A model can discriminate well in an external cohort while its explanation remains unstable; an explanation may also pass a technical test in a model that has not been externally validated. Visual plausibility does not establish faithfulness, and SHAP rankings can vary with the background data, correlated predictors, and preprocessing. Reports therefore need to identify the property being tested and the clinical setting in which the explanation is expected to be used (8–15).

Earlier medical-imaging reviews also found limited clinician involvement and inconsistent technical evaluation (15, 16). Van der Velden et al. (13) argued that evaluation should be matched to both the explanation and the clinical task. Arun et al. (14) further showed that widely used saliency methods could fail tests of localization, sensitivity, repeatability, or reproducibility. Bibliometric studies have documented rapid growth and concentration in major application domains (18, 22), whereas ultrasound-focused reviews have examined narrower oncological and organ-specific evidence (19, 21). A broader healthcare XAI review considered imaging alongside other applications (20). These studies did not combine cross-database coverage analysis with record-level explanation evaluation.

In ultrasound, this gap has an additional consequence: the acquisition itself can change both the model input and the explanation (3–6). Evaluation consequently extends to how the image was obtained as well as how the model was trained.

### 4.3 Acquisition-aware validation in ultrasound

In ultrasound, acquisition is part of the model system. The scanning plane, probe orientation and pressure, machine settings, frame selection, and acceptance of artifacts all shape the input presented to an algorithm. Speckle, acoustic shadowing, posterior enhancement, reverberation, anisotropy, and motion can carry diagnostic information, act as confounders, or do both, depending on the task (3–6). An explanation that is stable in one retrospective dataset may change when another operator or device acquires the same target.

Evaluation should therefore follow the acquisition pathway. Repeatability across model runs is different from stability across frames in a clip or sweep, and both are different from transportability across operators, devices, centers, and patient groups. Tests also need to distinguish perturbations that should preserve the clinical target from those expected to change the prediction. Prediction failures and explanation failures are most informative when examined together (3–6, 14, 49, 50).

Several practical tests can be built around routine ultrasound variation. If the target is unchanged, changes in gain, cropping, segmentation, or preprocessing can probe attribution stability. Adjacent frames can reveal sensitivity to frame selection. Held-out operators, devices, centers, and patient groups can then test transport beyond the development environment. A single visual inspection cannot cover these failure modes (14, 49, 50).

Relevant study designs already exist in ultrasound AI. Echocardiographic interpretation, multimodal breast risk, prenatal cardiac detection, and video-based cardiac function have been evaluated in stronger validation settings (40–43). Lung-ultrasound studies have examined classification and localization (44), while multicenter cohorts have been used for pediatric intussusception and liver applications (45, 46). Cross-center generalizability has also been studied in lung ultrasound (47), and noise-robustness testing has been proposed for ultrasound devices (48). What remains uncommon is a protocol that combines acquisition perturbation, external validation, and clearly defined explanation endpoints.

These examples establish the feasibility of repeated acquisitions, multicenter cohorts, video data, and prospective workflows, not the validity of any explanation. Predictive performance and explanation behavior remain separate outcomes, and both need their own endpoints.

Acquisition quality was the primary task in only four Core XAI records, despite its importance to both clinical interpretation and model performance. This finding suggests a potential role for explanations in flagging inadequate views, missing anatomy, or correctable acquisition problems during scanning (6). Such feedback would be more closely aligned with real-time acquisition than a heatmap produced after image capture.

### 4.4 From explanation generation to explanation evaluation

The practical starting point is a testable explanation claim. Localization can be checked against anatomical references, whereas stability can be examined under changes expected to preserve the clinical target.

Saliency-map randomization provides one such test: an explanation that remains visually similar after model parameters or labels are randomized is difficult to attribute to the learned function (49). Other studies have assessed localization, reproducibility, cross-method coherence, and disagreement between predictions and saliency maps (14, 17, 50). Representative failures should be specified before examples are selected.

Human evaluation was rarer still: 10 studies reported expert or reader assessment and three reported clinical-usefulness evaluation. A reader study should be designed around the decision that the explanation is intended to support. Depending on that role, the outcome may be diagnostic performance, reading time, confidence calibration, error detection, or a management decision. Studies in biliary atresia, fetal cardiac screening, and fetal growth assessment show that this type of clinician evaluation is feasible (51–53).

Confidence is a process outcome rather than a benefit in itself. Reader studies should examine it alongside accuracy, calibration, reading time, error detection, and the clinical decision that the explanation is intended to support.

Prospective workflow evaluation is warranted only when an explanation is intended to change behavior at the point of care. Retrospective data do not reproduce time pressure, incomplete acquisitions, real-time probe adjustment, competing clinical information, or the tendency to over-rely on an explanation. Technical and reader evaluation can precede prospective testing; post-deployment monitoring becomes relevant if the tool enters routine use (7, 54, 55).

### 4.5 Meaning of cross-database agreement and incremental yield

High rank concordance coexisted with substantial Scopus-only retrieval. Agreement among the leading entities therefore did not make either database sufficient for comprehensive retrieval.

The two databases also differ in source coverage, cited-reference completeness, and field structure. Combining native citation counts or reference networks can duplicate citation events and create links that reflect indexing rather than scholarship; searching only one source can miss relevant clinical or engineering journals. WoSCC science mapping was therefore kept database specific, while both databases were used for eligibility and coverage and source provenance was retained in comparisons of harmonized variables (26, 27).

Why a record appeared only in Scopus was not examined individually. Possible explanations include journal coverage, indexing timing, differences in affiliation and keyword fields, and the position of ultrasound XAI between clinical medicine and engineering (26, 27). The source-only counts show what a WoSCC-only search would have missed under the same eligibility rules. The rank correlations address a narrower question: whether the leading geographic and conceptual patterns were similar in the two database-specific datasets.

For annual, geographic, and conceptual features available in both databases, the leading patterns were similar despite meaningful differences in retrieval completeness. This separation preserved the internal coherence of citation-derived maps while making the additional coverage provided by Scopus explicit.

### 4.6 Strengths, limitations, and research implications

The review was designed to keep retrieval, science mapping, and evidence coding distinct. Two harmonized database searches addressed coverage; WoSCC supplied the cited-reference data used for mapping; and the Core set supported record-level coding. All unique records were screened independently by two reviewers and adjudicated by a third. Source provenance was retained throughout, and the frozen keyword thesaurus and bootstrap confidence intervals provided further checks. Partial-year 2026 data were excluded from complete-year temporal inference.

Coverage remains incomplete. Searches were limited to English-language articles and reviews in WoSCC and Scopus, and conference proceedings were excluded. Non-English studies, records indexed only in biomedical databases, and early engineering work may therefore be underrepresented. Citation counts are also sensitive to indexing practice and publication age, while changing XAI terminology may have produced false negatives.

Evidence coding depended mainly on indexed fields, with targeted full-text consultation when those fields were insufficient. Consequently, the reported proportions describe information identified in the reviewed evidence sources and may underestimate evaluation described only in unexamined full texts. Assigning one primary category also compresses hybrid studies, although Supplementary Data S1 retains record-level detail. No formal study-level methodological-quality or risk-of-bias assessment was performed because no single validated instrument was applicable across the heterogeneous tasks and designs; the four descriptive maturity indicators should not be interpreted as a substitute for such an assessment. Shared-list rank correlations may overstate agreement in the long tail, although union-list sensitivity results were similar. Corresponding-author-country counts do not describe training populations or deployment, and incomplete 2026 records contributed only to cumulative analyses. Although all records were independently screened and adjudicated, the complete item-level pre-adjudication decision matrix was not retained in an auditable form for retrospective calculation of an agreement statistic. Study endpoints were too heterogeneous for pooled diagnostic, reader, or explanation-effect estimates.

The immediate need is a testable explanation claim rather than a more elaborate display. Faithfulness and stability can then be examined under changes expected to preserve the clinical target. Studies intended to influence decisions should progress to reader or prospective multicenter workflow evaluation, with predictive performance and explanation behavior reported separately.

Negative results, representative failures, and the settings required to reproduce an explanation should be reported alongside successful examples, including preprocessing choices and explanation parameters that may alter the output (56). The accompanying reproducibility package provides public Python reconstruction scripts, software parameters and logs, version-27 thesauri, and non-proprietary derived source data.

## 5 Conclusion

Medical-ultrasound XAI is growing quickly and now spans many clinical domains. WoSCC and Scopus showed similar leading patterns, but reported explanation evaluation and clinically relevant validation remained limited. Future work should first define what an explanation is expected to show and how that claim will be tested. In ultrasound, such testing must account for the operator- and device-dependent acquisition process as well as the clinical workflow.

## Data Availability

The original contributions presented in the study are included in the article/Supplementary Material, further inquiries can be directed to the corresponding author.

## Supplementary material

Supplementary Figure S1 Domain-specific XAI methods, validation status, and reported study-design attributes in the WoSCC Core XAI dataset (*n* = 387). (A) Clinical domain by primary XAI method. Bubble area represents the number of records, and color represents the proportion with explicitly reported explanation evaluation. (B) Validation status and reported multicenter and prospective study attributes by clinical domain.

Supplementary Figure S2 Cross-database publication profiles and source coverage. (A) Normalized annual publication profiles in WoSCC and Scopus for complete years 2018–2025; the profiles were compared descriptively because the series were short and strongly time dependent. (B) Source composition of the Union Broad and Core XAI sets. Relative Scopus-only yield was 171/469 = 36.5% for Broad and 127/387 = 32.8% for Core XAI.
